# Human Umbilical Cord-Derived Mesenchymal Stem Cells Promote Corneal Epithelial Repair In Vitro

**DOI:** 10.3390/cells10051254

**Published:** 2021-05-19

**Authors:** Santhosh Kacham, Tejal Sunil Bhure, Sindhuja D. Eswaramoorthy, Gaurav Naik, Subha Narayan Rath, Sreenivasa Rao Parcha, Sayan Basu, Virender Singh Sangwan, Sachin Shukla

**Affiliations:** 1 Centre for Ocular Regeneration, Prof. Brien Holden Eye Research Centre, Sudhakar and Sreekanth Ravi Stem Cell Biology Laboratory, Hyderabad Eye Research Foundation, L.V. Prasad Eye Institute, Hyderabad 500034, India; santosh.biotech24@gmail.com (S.K.); tejalbhure7797@gmail.com (T.S.B.); gauravn07@gmail.com (G.N.); sayanbasu@lvpei.org (S.B.); drsangwan.lvpei@gmail.com (V.S.S.); 2 Department of Biotechnology, National Institute of Technology, Warangal 506004, India; 3 Indian Institute of Technology-Hyderabad, Hyderabad 502285, India; bm13p1004@iith.ac.in (S.D.E.); subharath@bme.iith.ac.in (S.N.R.); 4 The Cornea Institute, L.V. Prasad Eye Institute, Hyderabad 500034, India

**Keywords:** umbilical cord, mesenchymal stem cells, cornea, epithelium, regeneration, limbal stem cell deficiency

## Abstract

Corneal injuries are among the leading causes of blindness and vision impairment. Trauma, infectious keratitis, thermal and chemical (acids and alkali burn) injuries may lead to irreversible corneal scarring, neovascularization, conjunctivalization, and limbal stem cell deficiency. Bilateral blindness constitutes 12% of total global blindness and corneal transplantation remains a stand-alone treatment modality for the majority of end-stage corneal diseases. However, global shortage of donor corneas, the potential risk of graft rejection, and severe side effects arising from long-term use of immunosuppressive medications, demands alternative therapeutic approaches. Umbilical cord-derived mesenchymal stem cells can be isolated in large numbers using a relatively less invasive procedure. However, their role in injury induced corneal repair is largely unexplored. Here, we isolated, cultured and characterized mesenchymal stem cells from human umbilical cord, and studied the expression of mesenchymal (CD73, CD90, CD105, and CD34), ocular surface and epithelial (PAX6, WNT7A, and CK-8/18) lineage markers through immunofluorescence. The cultured human limbal and corneal epithelial cells were used as controls. Scratch assay was used to study the corneal epithelial repair potential of umbilical cord-derived mesenchymal stem cells, in vitro. The in vitro cultured umbilical cord-derived mesenchymal stem cells were plastic adherent, showed trilineage differentiation and expressed: mesenchymal markers CD90, CD105, CD73; epithelial marker CK-8/18, and ocular lineage developmental markers PAX6 and WNT-7A. Our findings suggest that umbilical cord-derived mesenchymal stem cells promote repair of the injured corneal epithelium by stimulating the proliferation of corneal epithelial cells, in vitro. They may serve as a potential non-ocular source of stem cells for treating injury induced bilateral corneal diseases.

## 1. Introduction

The cornea is the transparent part of the eye that helps in passing light to the retina. The transparency of the cornea is critical for optimal vision. However, genetic defects (e.g., Aniridia), ocular dryness, and traumatic injuries (e.g., thermal and chemical burns) compromise transparency and integrity of the cornea, leading to visual defects including blindness. Corneal injuries affecting epithelium and stroma may lead to corneal scarring, conjunctivalization, neovascularization, limbal stem cell deficiency, and sometimes complete blindness [1]. Corneal epithelium, the outermost layer of the cornea, is regenerated at regular intervals by limbal stem cells (LSCs). It maintains the corneal transparency, protects the eye against injury and infection, and aids the ocular immune response through production of inflammatory cytokines [2,3,4,5]. The loss of LSCs and their barrier function leads to limbal stem cell deficiency (LSCD), which is usually treated by transplantation of healthy LSCs from the contralateral eye [6,7,8,9]. However, bilateral complete LSCD patients lack autologous LSCs and are primarily treated by the transplantation of allogeneic LSCs, which may elicit an immune response leading to graft rejection [10,11]. An alternative approach for such bilateral corneal diseases could be using stem cells derived from non-ocular sources which have the ability to repair or regenerate injured limbal/corneal epithelium. Such cells could be mesenchymal stem cells (MSCs) [12,13], embryonic stem cells (ESCs) [14] or induced pluripotent stem cells (iPSCs) [15]; each of these have their own advantages and disadvantages. Multipotency and immunomodulatory properties of MSCs help in cellular differentiation and modulation of the immune response following injury. This makes them a better choice for cell therapy [16]. However, the differentiation of adult MSCs into corneal epithelial cells is challenging and has been explored for the last few years [17,18,19], with limited success [12,13,20].

Major sources of MSCs include bone marrow, adipose tissue, dental pulp, and corneal stroma. The isolation process from these sources is invasive and cell yield depends on the age of the donor. Another source without these limitations is the human umbilical cord. Umbilical cord-derived MSCs (UC-MSCs) are non-tumorigenic, less immunogenic, and relatively free of ethical concerns, and thus have the upper hand over other stem cell sources [21]. In addition to their trilineage differentiation (osteoblasts, chondroblasts, and adipocytes), UC-MSCs have been reported to differentiate into cardiomyocytes [22], neurons [23], oligodendrocytes [24], corneal endothelium [25] and hepatocytes [26].

The UC-MSCs are considered to be more primitive, proliferative and immunosuppressive than other MSCs [27] and there are only a handful of reports on corneal repair or regeneration by UC-MSCs [28,29,30,31,32,33]. Cords lining epithelial cells (CLECs) derived from umbilical cord amniotic membrane have been reported as a novel and promising source for ocular surface regeneration [28,29]. A Bioengineered CLEC-muc sheet, when transplanted in LSCD rabbit eyes, led to regeneration of a clear and smooth corneal surface with CK3^+^, CK12^+^, CK4^−^ and CK1/10^−^ phenotype [30]. Transplantation of MSCs derived from human neonatal umbilical cords onto thin and cloudy corneas of lumican null mice, resulted in significantly improved corneal transparency and increased stromal thickness (for congenital corneal diseases involving keratocyte dysfunction) [31]. MSCs derived from human umbilical cord blood home to injured corneal endothelium and are able to differentiate into human corneal endothelial-like cells ex vivo [25]. MSCs derived from human umbilical cord, when transplanted intrastromally, enabled host keratocytes to catabolize accumulated glycosaminoglycans and thus cured corneal defects (corneal clouding) in mucopolysaccharidosis (MPS) VII mice [32]. MSCs derived from human Whartson’s jelly have been shown to differentiate into corneal epithelial-like cells in a three-dimensional model in vitro [30].

Here, we first characterized the UC-MSCs isolated from whole human umbilical cord using explant culture method [34], studied their inherent capability to express molecules of ocular (PAX6 and WNT7A) and epithelial (CK8/18) lineage, and evaluated their role in corneal epithelial repair, in vitro. This is the first such study, to the best of our knowledge.

## 2. Materials and Methods

### 2.1. Culture of Human Umbilical Cord-Derived Mesenchymal Stem Cells (UC-MSCs)

The study was approved by the Institutional Review Board of the L.V. Prasad Eye Institute (LEC 04-15-039 and IC-SCRT-04-15-006), Hyderabad, India, and Institutional Ethical Committee of the Indian Institute of Technology, Hyderabad (IITH-IEC-2014-09-01). The UC-MSCs were isolated as described previously [34]. Three different samples were taken from females with an age range of 22–27 years with no history of systemic diseases like diabetes, cancer, or any other metabolic disorders for selection of the umbilical cord of their delivered babies. Briefly, the umbilical cord (obtained from patient with informed consent) was washed with antibiotic (1% Penicillin-Streptomycin)-containing phosphate buffered saline (PBS), cut into pieces of approximately 5 mm^3^ and placed in a petri dish. The explants were grown in Dulbecco’s Modified Eagle’s medium (DMEM) containing 10% fetal bovine serum (FBS), 2 mM L-glutamine, and 1% Penicillin-Streptomycin and maintained at 5% CO_2_ and 37 °C. All cell culture grade reagents were from Thermo Scientific Ltd., Waltham, MA, USA. The explants were left undisturbed for four days and the medium was changed twice weekly. The cells were grown in Cytomix medium (Miltenyi Biotec GmbH, Bergisch Gladbach, Germany) after subculture. The UC-MSCs used in this study belong to passage number 3 (P3).

### 2.2. Osteogenic, Adipogenic, and Chondrogenic Differentiation of UC-MSCs

The UC-MSCs were cultured in complete DMEM for three weeks along with osteogenic induction medium (consisting of 10 mM β-glycerophosphate (Sigma Aldrich, Saint Louis, MO, USA), 100 nM dexamethasone (Himedia, Mumbai, India), and 50 µg/mL l-Ascorbic acid (Himedia, Mumbai, India)), or adipogenic induction medium containing 1μM dexamethasone, 0.5 mM 3-isobutyl-l-methyl xanthine, 200 μM indomethacine, and 10 μg/mL of insulin, or chondrogenic induction medium [34]. After three weeks of culturing in osteogenic induction medium, alkaline phosphatase (ALP) staining was performed using ALP kit (Sigma-Aldrich, Saint Louis, MO, USA) according to the manufacturer’s protocol. After adipo-induction for four weeks, the UC-MSCs were stained with Oil Red O. Briefly; the cells were fixed with 10% formalin for 15 min and then incubated with 60% isopropanol for 5 min, following which the cells were stained with Oil Red O for 10 min. The petri plates were observed under microscope and images were taken. The chondrogenic assay was performed using Alcian blue staining as described by Eswaramoorthy et al. [34]. 

### 2.3. Culture of Human Limbal Epithelial Cells

Limbal epithelial cells were cultured in Human Corneal Epithelial (HCE) media prepared as previously described with slight modification (without Insulin like growth factor) [35]. Briefly, limbal biopsies were obtained from cadaveric donor corneas supplied by the Ramayamma International Eye Bank as per the protocol approved by the Institutional Review Board (LEC 04-15-039 and IC-SCRT-04-15-006). Mean age of the tissue samples used was 46 years. The inner surface of the corneal tissue was scraped using a surgical blade (No.21) to remove the residual endothelial cell layer. Superfluous corneal and scleral tissues were excised to isolate the limbal ring. To establish explant cultures, the isolated limbal ring was chopped into small pieces and placed on the culture plate and supplemented with HCE growth media containing Dulbecco’s modified Eagle’s medium (DMEM): Nutrient mixture F-12 (Gibco, Life Technologies Corporation, New York, NY, USA) supplemented with 10% fetal bovine serum (FBS) (HiMedia, Mumbai, India), 1× GlutaMAX (Gibco, Life Technologies Corporation, New York, NY, USA), 10 ng/mL human recombinant epidermal growth factor (hEGF) (HiMedia, Mumbai, India), and 1% Penicillin-Streptomycin (HiMedia, Mumbai, India), with regular media changes on alternate days for two weeks.

### 2.4. Culture of Corneal Epithelial Cells

Human corneal epithelial cells (HCE, P5) were kindly gifted by Araki-Sasaki’s group [36] and obtained from Dr. Indumathi Mariappan (L.V. Prasad Eye Institute, Hyderabad, India). The HCE cells were cultured with DMEM containing 10% fetal bovine serum (FBS), 2 mM L-glutamine, and 1% Penicillin-Streptomycin, and maintained at 5% CO_2_ and 37 °C with medium change twice a week. Alternatively, rabbit corneal epithelial cells (SIRC; Statens Seruminstitut Rabbit Cornea, ATCC^®^ CCL-60™) [37,38,39] were procured from the national repository at the National Centre for Cell Sciences, Pune, India.

### 2.5. Immunofluorescence

Human limbal epithelial (LECs), corneal epithelial (HCE), umbilical cord-derived MSCs (UC-MSCs), and rabbit corneal epithelial cells (SIRC) were cultured on glass coverslips in 12-well plates and fixed in 4% paraformaldehyde (Sigma Aldrich, Saint Louis, MO, USA) solution for 10 min at room temperature. The cells were then permeabilized using a 0.5% Triton X-100 (Sigma Aldrich, Saint Louis, MO, USA) solution for 10 min at room temperature. Blocking to prevent nonspecific antibody binding was achieved with 2% Bovine Serum Albumin (Sigma Aldrich, Saint Louis, MO, USA). Primary antibody (Table 1) incubation (1:100 dilution) was carried out overnight at 4 °C, followed by incubation with species-specific fluorochrome-conjugated secondary antibody (Table 2) for 45 min. After incubation with primary and secondary antibodies, cells were washed with 1× PBS (Phosphate Buffered Saline: 137 mM NaCl, 2.7 mM KCl, 10 mM Na_2_HPO, and 1.8 mM KH_2_PO_4_) 3 times for 15 min each. Cells were mounted in DABCO™ (Abcam, Cambridge, MA, USA) (cell side down) and observed under an inverted fluorescence microscope (Scope.A1 AX10, Carl Zeiss, Jena, Germany). The primary and secondary antibodies used in immunofluorescence are shown in Table 1 and Table 2, respectively. The immunofluorescence staining was repeated at least three times and specificity of the antigen was determined using negative controls for primary and secondary antibodies, respectively.

### 2.6. Flow Cytometry

UC-MSCs were suspended to form a single cell suspension and stained with fluorochrome-conjugated monoclonal antibodies and appropriate isotype controls [34]. Briefly, culture medium was removed; UC-MSCs were washed with 1× PBS (free from Ca^++^ and Mg^++^), collected through scrapper and suspended in 1× PBS. 1 × 10^6^ UC-MSCs were mixed with 2 mL of 1× FACS buffer (1× PBS supplemented with 2% FBS) and centrifuged at 300× *g* for 5 min at 4 °C. Pellet was suspended in 100 μL of FACS buffer, respective antibodies (CD45 (#A07783), CD34 (#IM1870), CD73 (#B68176), CD90 (#B36121), and CD105 (#B76299) from Beckman Coulter, Inc. (Brea, CA, USA) were added as per manufacturer’s instructions and mixture was incubated at 4 °C for 45 min. Following antibody incubation, UC-MSCs were washed with 1 mL FACS buffer, centrifuged at 300× *g* for 5 min at 4 °C, pellet was suspended in 500 μL FACS buffer and stained cells were analyzed on CytoFLEX flow cytometer (Beckman Coulter, Inc., Brea, CA, USA). The data were analyzed using CytExpert software (Beckman Coulter, Inc., Brea, CA, USA). 

### 2.7. In Vitro Scratch Assay

The corneal epithelial cells of rabbit (SIRC) and human (HCE) origin were seeded in 12-well plates and allowed to grow till complete confluency to form a monolayer. A wound/scratch passing through the diameter of the well was created in the center with the help of a T200 pipette tip and the monolayer was washed and replenished with fresh medium to remove the debris. UC-MSCs (1 × 10^5^ cells per insert) were added into 0.4 μm culture inserts (TCP084, HIMEDIA Laboratories, Mumbai, India) and placed over the wounded corneal epithelial cells. Wounded corneal epithelial cells without any contact with MSCs were used as controls (mock). For conditioned medium, (UC-MSC-CM or CM) UC-MSCs were allowed to grow in a T25 flask till 70–75% confluency, medium was aspirated and fresh medium (DMEM-F12 supplemented with 10% FBS and 1% penicillin-streptomycin) was added and incubated for the next 24 h. CM was collected, centrifuged at 500× *g* for 5 min to remove cell debris, and the supernatant was used for the scratch assay experiment. The wounded corneal epithelial cells incubated with the conditioned medium derived from UC-MSCs (UC-MSC-CM) were used as an alternate control. Epithelial repair was studied through regular microscopic observations at defined intervals (0, 24, 48, and 72 h for SIRC; and 0, 12, 24, and 36 h for HCE) and images were captured and analyzed through ImageJ software (version: 64-bit, Java 1.8.0_172) (https://imagej.nih.gov/ij/).

To determine whether the effect of UC-MSCs on the scratch wound was related to migration or proliferation of corneal epithelial cells, HCE and SIRC, at complete confluency, were treated with 10 μg/mL of Mitomycin C (Zydus Healthcare Ltd., Ahmedabad, India) for 3 h at 37 °C, washed three times with PBS, and used for scratch assay as mentioned above.

### 2.8. Statistical Analysis

The results are based on three independent sets of experiments in triplicates. The data is presented as mean ± SD. Statistical significance (*p* < 0.05) was determined by Student’s *t*-test.

## 3. Results

### 3.1. Culture and Characterization of Cells

**Figure 1:**
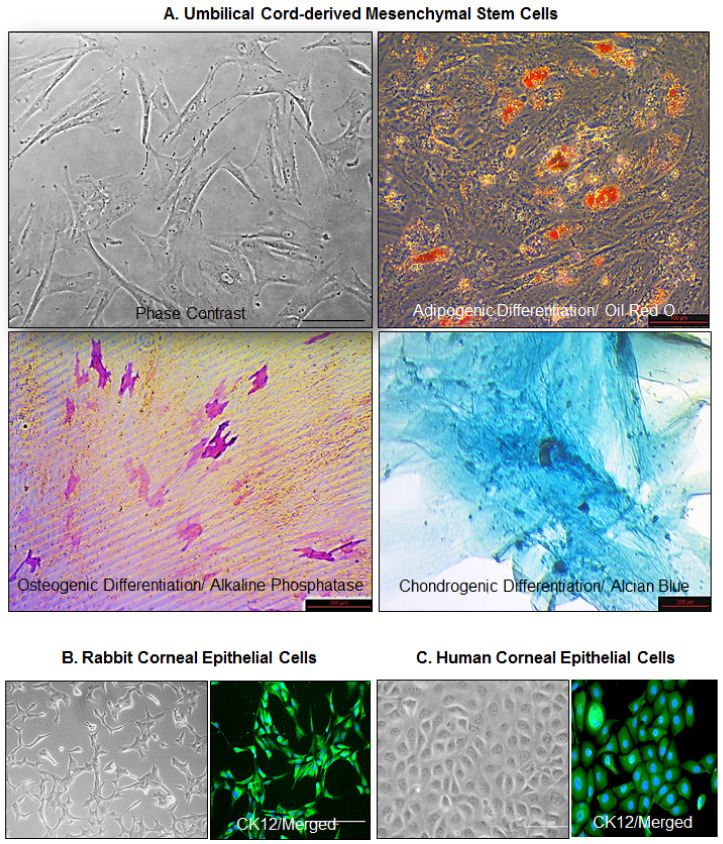
Culture and characterization of cells. (**A**) Umbilical cord-derived mesenchymal stem cells (UC-MSCs) were cultured in Cytomix medium (Miltenyi Biotec GmbH, Bergisch Gladbach, Germany) and their adipogenic, osteogenic, and chondrogenic differentiation was studied using Oil Red O, Alkaline Phosphatase, and Alcian Blue, respectively. (**B**) Rabbit corneal epithelial cell line (SIRC) and (**C**) Human corneal epithelial (HCE) cells were characterized by expression of CK12. Bar size: 100 μm.

The phase contrast microscopic observations revealed that the cultured UC-MSCs were plastic adherent, elongated and fibroblastic in shape (Figure 1A). Their differentiation into adipocytes, osteoblasts, and chondroblasts, in vitro, was checked through Oil red O, Alkaline Phosphatase (ALP), and Alcian Blue assays, respectively. The Oil Red O staining showed that the cells could differentiate into adipocytes; red color indicates the staining of the lipid deposits (Figure 1A). The ALP staining showed that the UC-MSCs differentiated into osteoblasts (red/purple), whereas Alcian Blue staining revealed their differentiation into chondroblasts. This validated their trilineage differentiation potential, characteristic of mesenchymal stromal cells (Figure 1A).

The epithelial cells, on the other hand, were hexagonal and cuboidal in shape. The rabbit corneal epithelial cells (Figure 1B) and HCE cells (Figure 1C) were characterized by expression of CK12, a marker of differentiated corneal epithelial cells. 

### 3.2. Expression of Mesenchymal Stem Cell Markers

**Figure 2:**
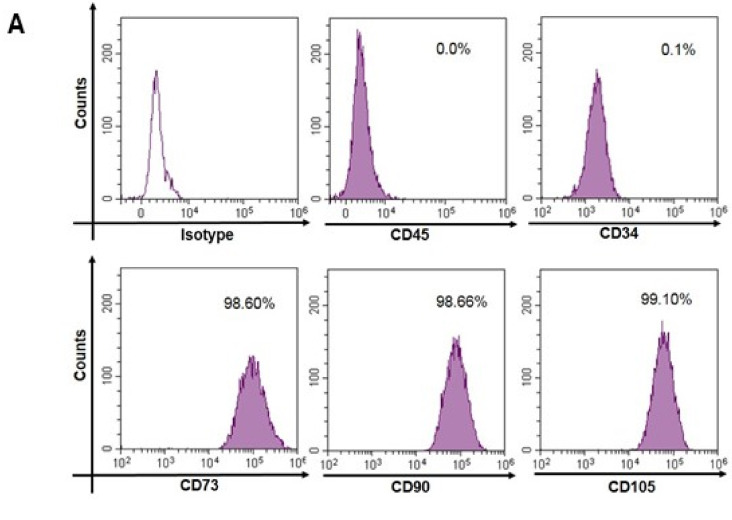

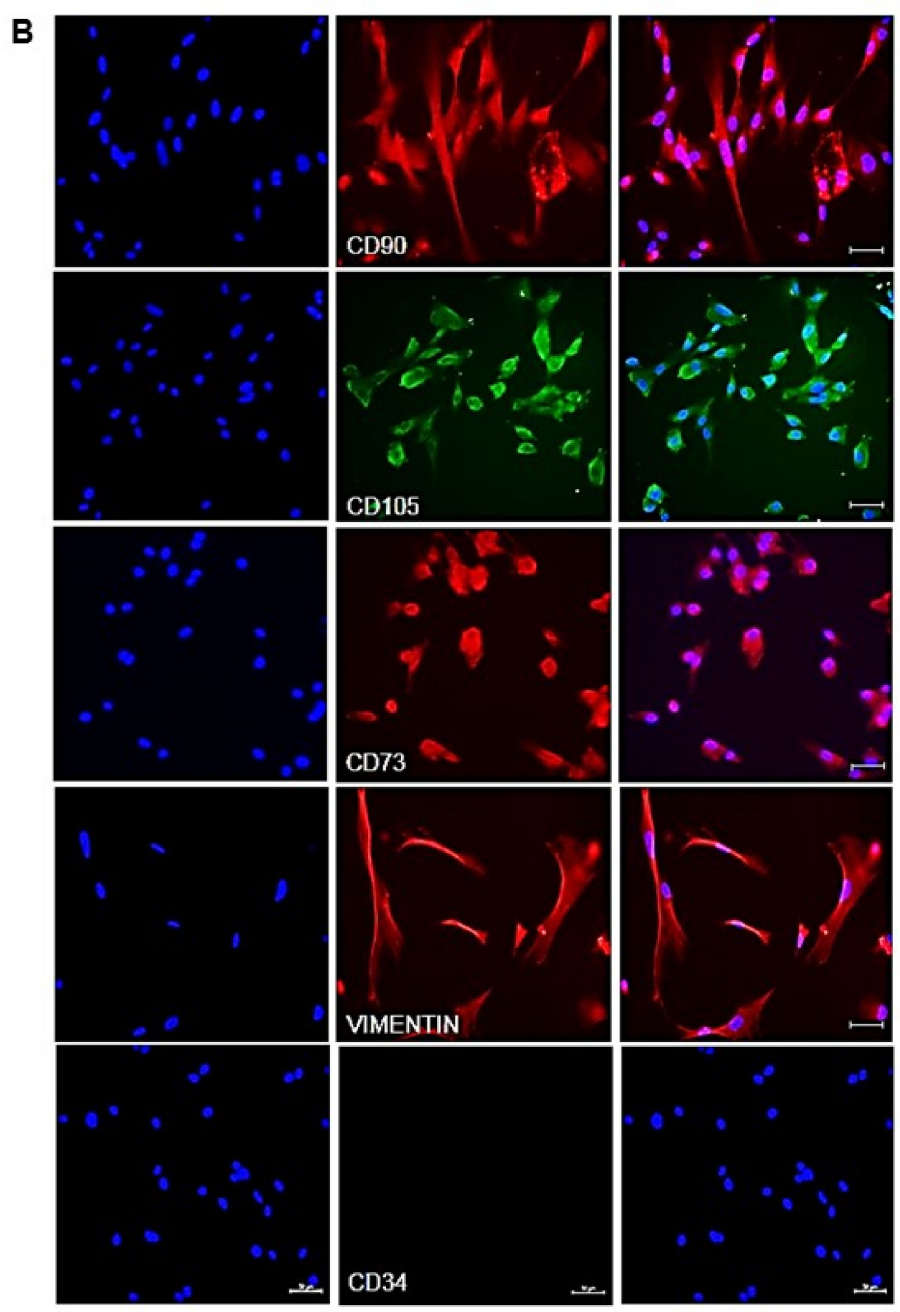
Expression of mesenchymal markers by the human umbilical cord-derived mesenchymal stem cells (UC-MSCs). (**A**) The cultured UC-MSCs showed negative expression of CD45 and CD34 but positive expression of CD73, CD90, and CD105, in flow cytometry. (**B**) Immunofluorescence was used to study the expression and cellular localization of CD90, CD105, CD73, VIMENTIN, and CD34. The left panel shows DAPI staining, the middle panel shows antigen specific staining and the right panel shows merged image of both DAPI and antigen specific staining. Bar size: 50 μm.

The International Society for Cellular Therapy (ISCT) in a position statement recommended that MSCs must express CD105, CD73, and CD90, and lack expression of CD34 and CD45 [40]. Accordingly, the cultured UC-MSCs were characterized using flow cytometry and immunofluorescence for studying expression of these different markers. All the cultured UC-MSCs expressed the characteristic MSC markers CD90, CD105, CD73, and VIMENTIN, whereas expression of CD34 and CD45 was undetectable in these cells. (Figure 2A,B). The flow cytometry analysis revealed that 98.6% cells were positive for CD73, and CD90 whereas 99.1% cells were positive for CD105 (Figure 2A). The quantitative analysis of these cells is also described by Eswaramoorthy et al. [34].

### 3.3. Expression of Ocular Lineage Markers by UC-MSCs

**Figure 3:**
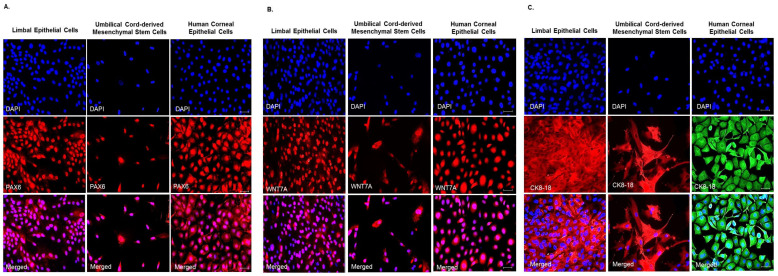
In vitro cultured human umbilical cord-derived mesenchymal stem cells (UC-MSCs) show endogenous expression of (**A**) PAX6, an ocular surface developmental marker [40] and a pleiotropic master regulator of eye development [41,42], (**B**) WNT7A, a key signaling molecule of the Wnt pathway which controls proliferation of human corneal epithelial stem cells [43], and (**C**) CK-8/18, an epithelial marker [44] under standard culture conditions. This indicates that the hUC-MSCs may possess inherent potential for corneal epithelial repair/regeneration. The human limbal and corneal epithelial cells were used as controls. The top panel shows DAPI staining, the middle panel shows antigen specific staining and the lower panel shows merged image of both DAPI and antigen specific staining in each of the three cell types, respectively. The specificity of the staining was determined by suitable isotype controls (data not shown). Bar size: 50 μm.

The WNT7A is reported to regulate corneal epithelial differentiation through the transcription factor PAX6. The WNT7A-PAX6 axis has a central role in fate determination of corneal epithelial cells [41]. To study the inherent potential of UC-MSCs to differentiate into cells of corneal epithelial lineage, we studied the expression of PAX6, WNT7A and CK8/18 in the UC-MSCs, corneal (HCE) and limbal epithelial (LECs) cells through immunofluorescence. UC-MSCs, along with HCE and LECs, showed the expression of ocular surface developmental marker PAX6 (Figure 3A), signaling molecule WNT-7A (Figure 3B), and epithelial marker CK8/18 (Figure 3C). 

### 3.4. Role of UC-MSCs in Injury-Induced Corneal Epithelial Repair

**Figure 4:**
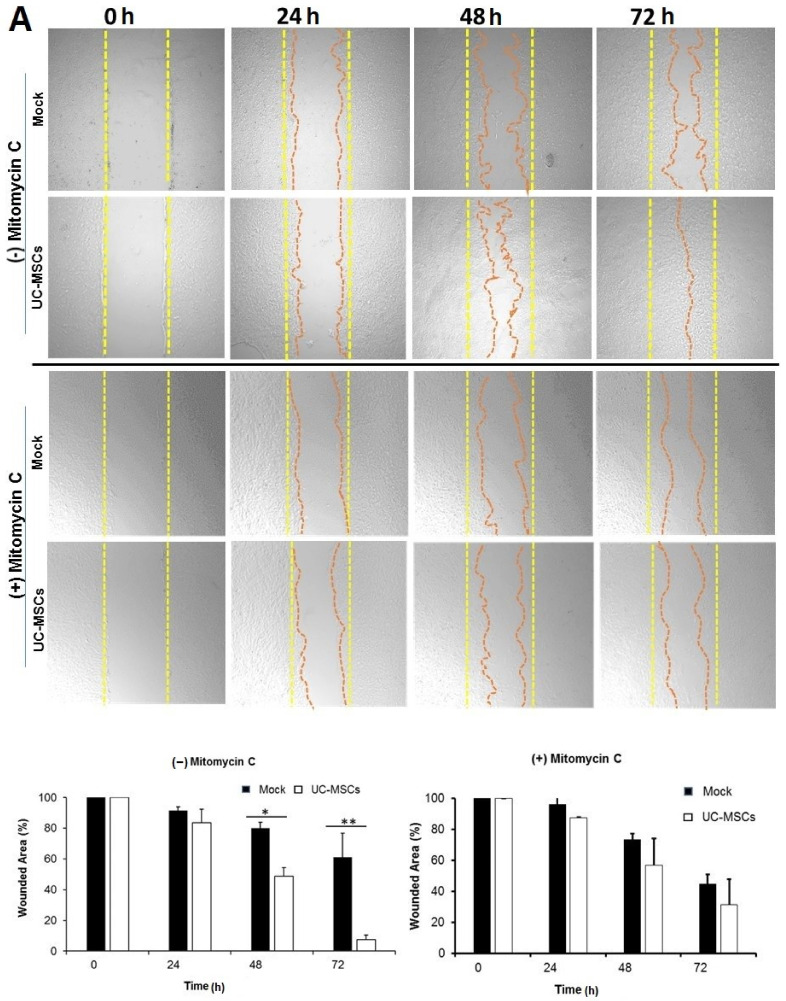

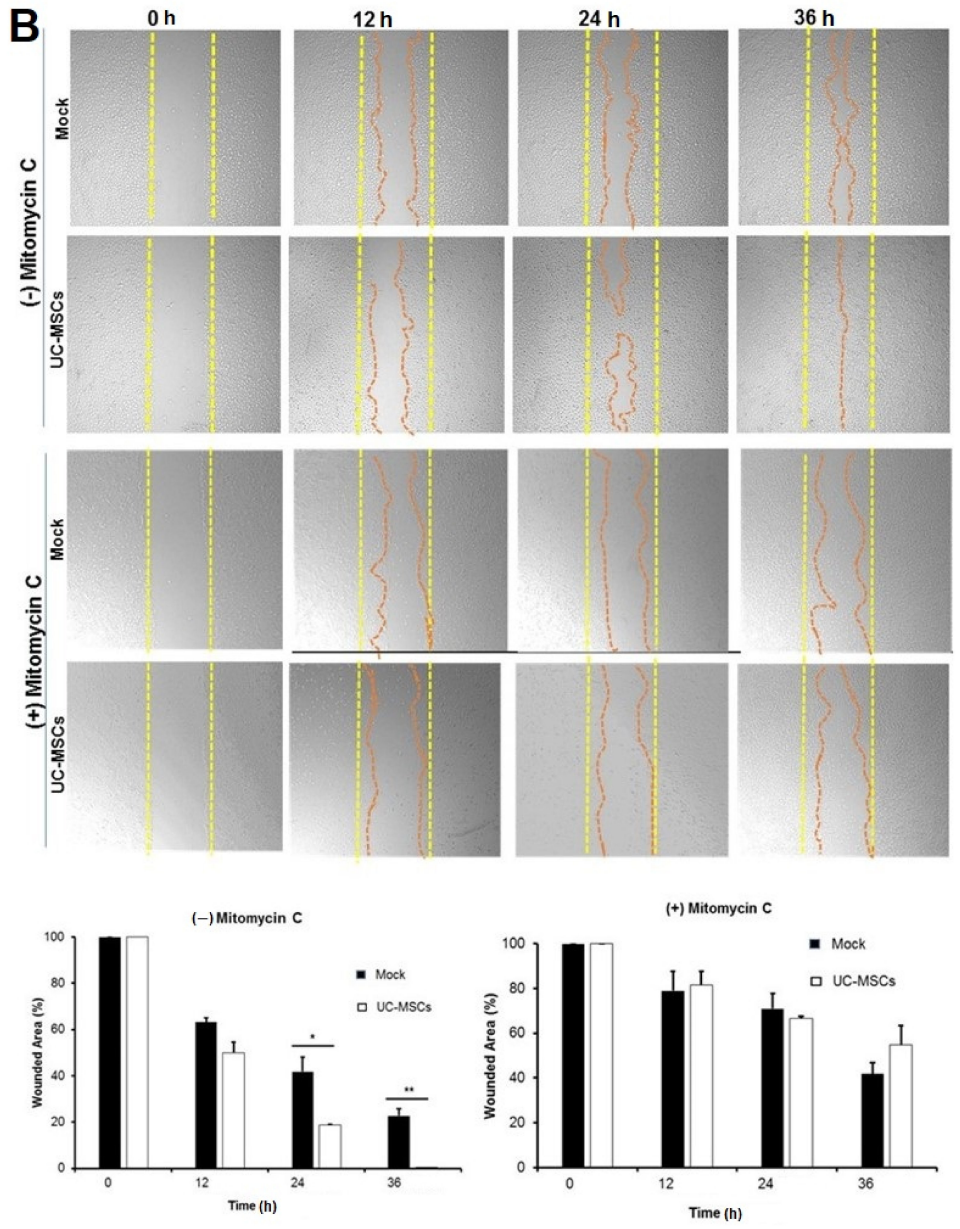
Umbilical cord-derived mesenchymal stem cells accelerate corneal epithelial repair by stimulating the proliferation of corneal epithelial cells in vitro. Scratch assay was used as an in vitro model to study the epithelial repair potential of UC-MSCs in cultured (**A**) rabbit (SIRC) and (**B**) human (HCE) corneal epithelial cell lines. Mitomycin C treatment (10 μg/mL for 3 h, prior to scratching) was used to inhibit the proliferation of corneal epithelial cells. To mimic epithelial injury, a completely confluent corneal epithelial monolayer was wounded through scratching. Scratched monolayer was incubated with UC-MSCs maintaining indirect contact through culture inserts. Wounded corneal epithelial cells without any incubation with MSCs were used as controls (mock). Epithelial repair was studied through microscopic observations at defined intervals (0, 24, 48, and 72 h in SIRC, and 0, 12, 24, and 36 h in HCE). Total area of the wound/scratch at 0 h was expressed as 100%. Representative data from three independent experiments are shown and values are expressed as mean ± SD (error bar). * *p* < 0.05, ** *p* < 0.01.

MSCs are reported to have tissue repair capabilities. To evaluate the role of UC-MSCs in the healing of the wounded corneal epithelium, scratch assay was performed with rabbit (SIRC; Figure 4A) and human (HCE, Figure 4B) corneal epithelial cells, in vitro. A scratch mimicking corneal epithelial injury was made in a completely confluent monolayer of cultured corneal epithelial cells and UC-MSCs were analyzed for their repair function by establishing indirect contact with scratched corneal epithelial monolayer through culture inserts. The scratch was followed up till complete closure, post-injury (scratching). The scratched monolayer without any contact with UC-MSCs was used as control (mock). The area of the scratch was calculated through ImageJ and expressed as a percentage. The total area of the scratch at 0 h was considered as 100%. Relative to mock, UC-MSCs significantly accelerated wound closure in rabbit (at 48 (80.11 ± 3.82% vs. 48.78 ± 5.57%) and 72 h (60 ± 15.58% vs. 7.64 ± 2.68%), (Figure 4A)), and human (at 24 (41.81 ± 6.25 vs. 18.75 ± 4.47), and 36 h (22.66 ± 2.93 vs. 0.32 ± 0.1), (Figure 4B)) corneal epithelial cells, respectively, in terms of scratched area remaining for closure. In rabbit corneal epithelial cells (SIRC), at 72 h, UC-MSCs significantly accelerated wound closure with only 7% (7.64 ± 2.68%) of the scratched area remaining, relative to mock, which still had 60% (60 ± 15.58%) of the scratched area remaining to be repaired/closed (Figure 4A). In comparison, in HCE, at 36 h, UC-MSCs had only 0.3% (0.32 ± 0.1) of the scratched area remaining, relative to mock, which still had 22% (22.66 ± 2.93) of the scratched area remaining for closure (Figure 4B).

On the other hand, Mitomycin C-treated corneal epithelial cells showed significantly reduced rate of wound closure compared to untreated cells. Moreover, addition of UC-MSCs to Mitomycin C-treated epithelial cells couldn’t change the rate of wound closure significantly (Figure 4A,B). Relative to mock (Mitomycin C-treated corneal epithelial cells), UC-MSCs showed increase in rate of epithelial wound closure in SIRC (at 24 (96.21 ± 11.73% vs. 87.53 ± 1.5%), 48 (73.47 ± 3.94% vs. 56.78 ± 17.53%) and 72 h (44.90 ± 5.98% vs. 31.62 ± 16.44)) and HCE (at 24 h (71 ± 6.73% vs. 66.7 ± 9.3%)) cells (Figure 4A,B), respectively.

## 4. Discussion

The umbilical cord is among the foremost fetal tissues explored so far for presence of stem cells [45]. It is one of the supportive extra embryonic tissues including amniotic fluid and placenta, which is typically discarded as biological waste, postpartum. Being of newborn origin, the umbilical cord-derived stem cells are immunologically naïve, less immunogenic, non-tumorigenic, and have excellent proliferation and differentiation potential for tissue repair, following injury [45,46]. In current clinical practice, the umbilical cord-derived stem cells are stored for the neonates, who can be a source of autologous stem cells for unforeseen events in their adult life [47,48], and can also be used in allogeneic settings, if required. Owing to these properties, UC-MSCs may evolve as the new gold standard for MSC-based therapies in the near future [27].

This is the first study, to the best of our knowledge, which has shown the effectiveness of UC-MSCs derived from explant culture of whole human umbilical cord, for corneal epithelial repair following injury, in vitro. Here, the expression of CD73, CD90, CD105 and VIMENTIN by hUC-MSCs validates their mesenchymal origin and is in accordance with earlier reports [49]. Their differentiation into adipocytes, osteoblasts and chondroblasts when cultured in respective induction media for three weeks further confirms their stemness. In vitro cultured UC-MSCs expressed ocular surface developmental marker PAX6 [40] and signaling molecule WNT7A [43], which indicates their inherent potential for differentiation into ocular phenotypes. When analyzed for epithelial markers, the cultured UC-MSCs expressed simple epithelial marker CK-8/18. The differentiation state of UC-MSCs (partial or complete) may also be critical for expression of markers.

Earlier published reports have derived MSCs largely from different parts of the umbilical cord, i.e., cord blood [25], Whartson’s jelly [22,24,30], and amniotic membrane [28,29] and shown that the UC-MSCs express various epithelial markers; however there has been no such previous report on explant culture of whole umbilical cord to derive MSCs, to the best of our knowledge [33]. Reza et al. earlier showed that the umbilical cord lining cells which specifically express MUCIN1 (CD277) were able to differentiate into corneal epithelial cells under specific culture conditions [28]. The novelty of this study lies in derivation of UC-MSCs from whole umbilical cord using simple explant culture method and further evaluation of their ability of corneal epithelial repair using an in vitro scratch assay model. 

Initially, we checked whether in standard culture conditions the cultured UC-MSCs endogenously express epithelial marker CK-8/18 [44]. Further, we checked the endogenous expression of the PAX6, a master regulator of eye development [50,51] a marker of ocular surface development [40] and multi-level regulator of ocular development including corneal epithelial homeostasis [41,42,52] and signaling molecule WNT7A [43] by UC-MSCs and compared them with HCE and LECs, in normal culture conditions. Ouyang H et al. studied the crucial role of homeostasis between PAX6 and WNT7A in the differentiation of the corneal epithelial cells [52] and a recent study reports that PAX6 induces differentiation of rat adipose-derived MSCs into corneal epithelial cells [53]. PAX6 and WNT7A are reported to regulate corneal epithelial homeostasis and play a key role in determining the fate of the corneal epithelial cells and in specification of the corneal lineage [52]. While UC-MSCs are largely unexplored for their corneal differentiation potential, bone marrow-derived MSCs (BM-MSCs) are reported to reduce corneal opacity, accelerate corneal epithelial repair, and suppress inflammation in a murine model of corneal injury [54]. 

UC-MSCs, LECs and HCE were positive for the ocular surface marker PAX6 (>90% positive cells in all LE, UC-MSCs and HCE) and signaling molecule WNT7A (71% in LE, 89% in UC-MSCs, and 95% in HCE) (Figure 3A,B). The differences in their numbers and distribution in a given microscopic field are likely due to differences in their morphology (UC-MSCs in culture are elongated, fibroblastic, and dispersed whereas corneal and limbal epithelial cells are hexagonal and densely packed). Expression of PAX6 and WNT7A by the UC-MSCs indicates that these cells may intrinsically possess the potential to differentiate into cells of ocular lineage including corneal epithelial cells [55]; however, the nature of factors required to do so is a matter of further investigation. Their differentiation into corneal epithelial cells is possibly easier in direct in vivo applications due to the presence of corneal niche and surroundings as compared to an in vitro setting. 

Our results show that hUC-MSCs accelerate corneal epithelial repair in in vitro models of corneal epithelial injury using human (HCE) and rabbit (SIRC) corneal cell lines. However, the conditioned medium derived from UC-MSCs couldn’t show a significant acceleration of epithelial wound closure (Supplementary Figure S1A,B). Further treatment of HCE and SIRC with Mitomycin C, a known potential inhibitor of DNA synthesis and cell proliferation, significantly reduced the rate of UC-MSCs-induced epithelial repair. This observation led us to conclude that UC-MSCs accelerate corneal epithelial wound closure primarily by promoting proliferation of corneal epithelial cells in vitro. This may also be attributed to the extracellular vesicles/exosomes secreted by UC-MSCs. The underlying mechanism is subject of further investigations. MSCs have also been reported to stimulate the proliferation of limbal stem cells and native corneal cells [56]. MSC-secreted growth factors are required for the proliferation and migration of corneal epithelial cells, and they contribute to the regeneration of corneal epithelium [57,58]. We strongly feel that scratch assay can be used as an in vitro model of corneal repair in aforesaid conditions. Since mesenchymal stem cells are usually hypoimmunogeneic, the human MSCs have also been applied to animal (rabbit/mouse) models of corneal injury [59]. We further propose that administration of these UC-MSCs in corneal epithelial injury in vivo may accelerate epithelial wound closure through paracrine factors or through direct differentiation. In addition, endogenous expression of PAX6 and WNT7A by these cells suggests their potential for differentiation into corneal epithelial like cells. However, we understand that these are subjected to future studies and if successful, this would further support the potential of these cells to be used as an alternative stem cell source specifically in the treatment of bilateral corneal defects, where autologous corneal/limbal stem cells are not available for transplantation [60]. A proposed clinical trial (NCT03237442, Umbilical Cord Mesenchymal Stem Cells Injection for Ocular Corneal Burn, https://clinicaltrials.gov/ct2/show/NCT03237442, accessed on 7 May 2021) by Guangzhou Saliai Stem Cell Science and Technology Co. Ltd. (Guangzhou, China) may yield promising results.

We understand the limitations of our study and acknowledge that sequencing of the cell lines used here, and their detailed authentication and characterization would have consolidated our findings. The phenotypic characterization of rabbit corneal epithelial cell line (SIRC) suggests their mixed epithelial and fibroblastic nature [39]. Being an in vitro study, cell-based variations cannot be ignored and translational applications of these UC-MSCs would require further validation and assessment of their safety and efficacy through in vivo animal studies.

## 5. Conclusions

In conclusion, our results demonstrate that human umbilical cord-derived mesenchymal stem cells promote corneal epithelial repair.

## Figures and Tables

**Table 1:** List of primary antibodies used in immunofluorescence.

S. No.	Antibody	Supplier	Catalogue/Clone	Concentration *	Dilution
1	CK-8/18 Mouse monoclonal	Santacruz	sc-52325/NCL-5D3	200 µg/mL	1:100
2	CD-34 Mouse monoclonal	Santacruz	sc-7324/ICO115	200 µg/mL	1:100
3	CD-73 Rabbit monoclonal	Cell Signaling	#13160/D7F9A	155.2 µg/mL	1:100
4	CD-90 Mouse monoclonal	Santacruz	sc-59396/AF-9	100 µg/mL	1:100
5	CD-105 Mouse monoclonal	Santacruz	sc-376381/A-8	200 µg/mL	1:100
6	PAX-6 Rabbit polyclonal	Santacruz	sc-11357/H-295	200 µg/mL	1:100
7	WNT-7A Mouse monoclonal	Santacruz	sc-365665/E-9	200 µg/mL	1:100
8	VIMENTIN Mouse monoclonal	Santacruz	sc-6260/V9	200 µg/mL	1:100

Suppliers: Santacruz Biotechnology Inc., Dallas, TX, USA.; Cell Signaling Technology, Danvers, MA, USA; * Stock Concentration.

**Table 2:** List of Secondary Antibodies used in Immunofluorescence.

S. No.	Antibody	Supplier/Catalogue	Concentration *	Dilution
1	Alexa flour^®^ 488 Goat Anti-Mouse IgG	Abcam/ab150113	2 mg/mL	1:400
2	Alexa Fluor^®^ 594 Goat Anti-Mouse IgG	Abcam/ab150120	2 mg/mL	1:400
3	Alexa Fluor^®^ 488 Goat Anti-Rabbit IgG	Abcam/ab150077	2 mg/mL	1:400
4	Alexa Fluor^®^ 594 Goat Anti-Rabbit IgG	Abcam/ab150080	2 mg/mL	1:400

Supplier Details: Abcam, Cambridge, MA, USA. * Stock Concentration.

## Data Availability

Data supporting reported results can be obtained from the corresponding author (sachin@lvpei.org) and Subha N. Rath (subharath@bme.iith.ac.in).

## Supplementary Materials

The following are available online at https://www.mdpi.com/article/10.3390/cells10051254/s1, Figure S1: The conditioned medium derived from umbilical cord-derived mesenchymal stem cells (UC-MSCs) couldn’t show a significant acceleration of corneal epithelial wound closure.
