# Supplementary material for: Human Umbilical Cord-Derived Mesenchymal Stem Cells Promote Corneal Epithelial Repair In Vitro
Source: Cells. 2021 May 19;10(5):1254. doi: 10.3390/cells10051254 (PMC8160941; doi:10.3390/cells10051254)
Supplement: Supplementary file 1 [file cells-10-01254-s001.zip › cells-1153524-supplementary.pdf]

cells MDPI

Supplementary Material

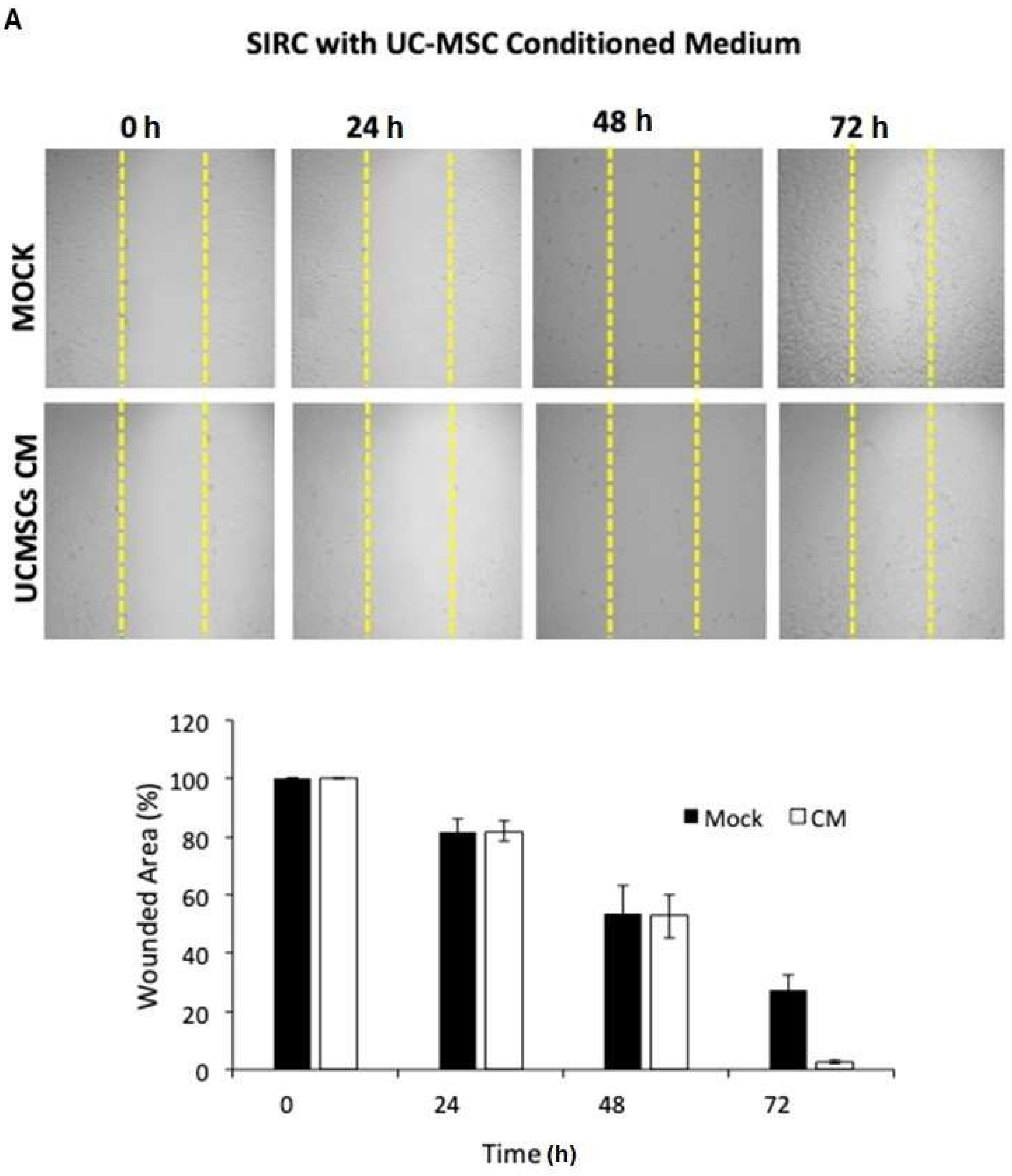

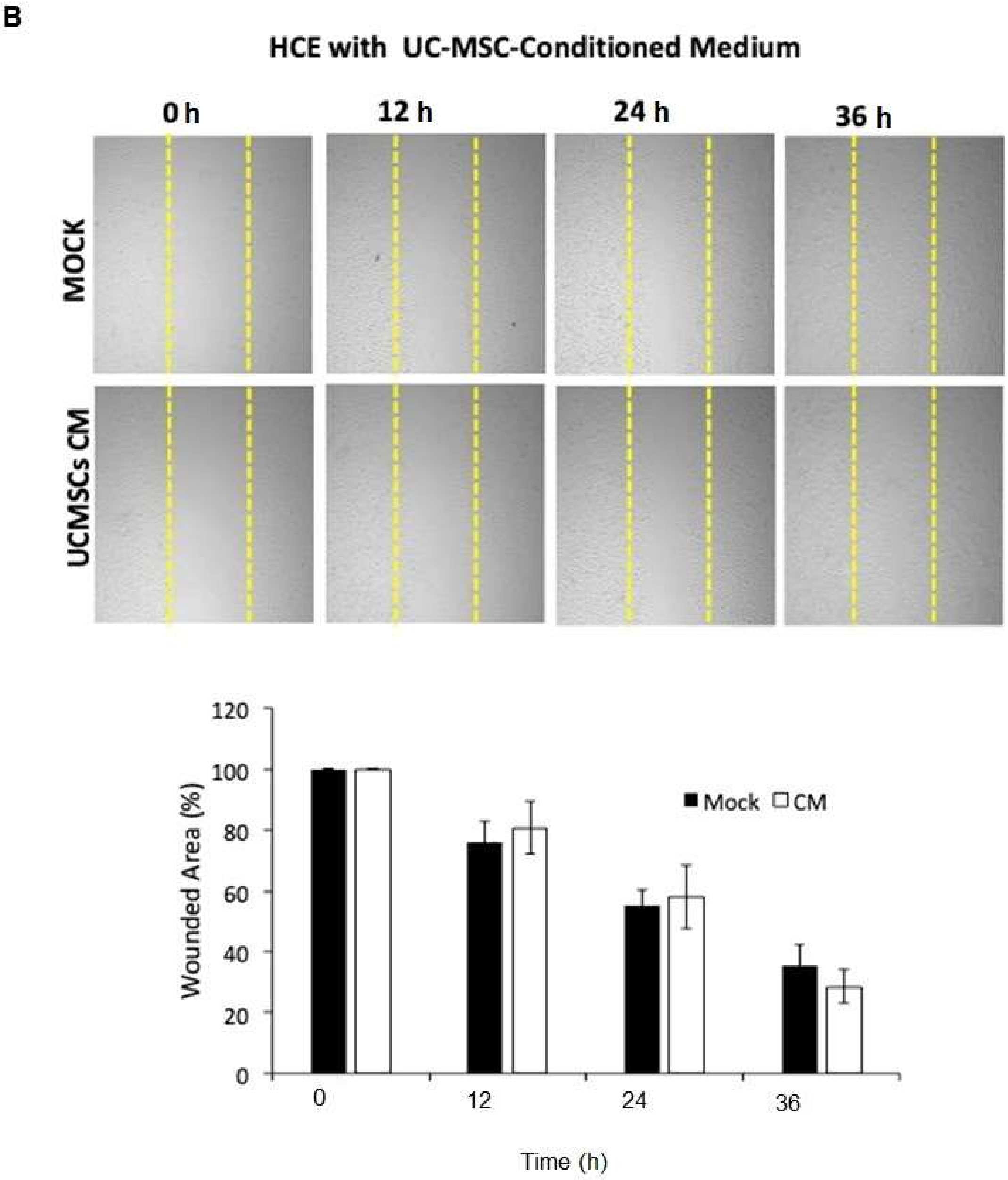

Supplementary Figure S1. The conditioned medium derived from umbilical cord-derived mesenchymal stem cells (UC-MSCs) couldn't show a significant acceleration of corneal epithelial wound closure. To mimic epithelial injury, completely confluent corneal epithelial monolayer was wounded through scratching. For conditioned medium (CM) UC-MSCs were allowed to grow in T25 flask till 70–75% confluency and then the media was aspirated and new DMEM F12 + 10% FBS with antibiotics was added and incubated for 24 h. CM was collected and centrifuged at 500× *g* for 5 min to remove cell debris and the supernatant was used as CM for the scratch experiments. Wounded corneal epithelial cells without any incubation with CM were used as controls (mock). Epithelial repair was studied through microscopic observations at defined intervals in (**A**) SIRC (0, 24, 48, and 72 h) and (**B**) HCE (0, 12, 24, and 36 h). Total area of the wound/scratch at 0 h was expressed as 100%. Representative data from three independent experiments are shown and values are expressed as mean ± SD (error bar).
